# Dietary Inflammatory Index and diabetic retinopathy risk in US adults: findings from NHANES (2005–2008)

**DOI:** 10.1186/s12886-024-03303-1

**Published:** 2024-01-30

**Authors:** Rong Liu, Jiechang Zhang, Wen Gu, Xiujuan Zhao, Lishun Xiao, Chengcheng Yang

**Affiliations:** 1 Department of Biostatistics, School of Public Health, Xuzhou Medical University, 209 Tongshan Road, 221004 Xuzhou, China; 2 Department of Ophthalmology, The Fifth Affiliated Hospital of Sun Yat-sen University, 52 Meihua Road, 519000 Zhuhai, China; 3 Department of Cardiology, Zhuhai People’s Hospital, Zhuhai, China; 4 State Key Laboratory of Ophthalmology, Zhongshan Ophthalmic Center, Guangdong Provincial Key Laboratory of Ophthalmology and Visual Science, Guangdong Provincial Clinical Research Center for Ocular Diseases, Sun Yat-sen University, Guangzhou, China

**Keywords:** Diabetic retinopathy, Dietary inflammatory index, Nutrition, Food consumption, NHANES

## Abstract

**Background:**

Inflammation is associated with the pathophysiology of diabetic retinopathy (DR). Within the framework of complete dietary patterns, the Dietary Inflammatory Index (DII) was formulated to evaluate the inflammatory properties inherent in a diet. The main purpose of the current study was to assess the relationship between DII and DR using National Health and Nutrition Examination Survey (NHANES).

**Methods:**

The original sample size included 1,148 diabetes patients out of 2005–2008 NHANES surveys. Twenty-four-hour dietary consumptions were used to calculate the DII scores. Demographic characteristics and retina examinations were collected for the comparison between DR and non-DR groups in diabetes patients. The relationship between DII and DR was analyzed by a logistic regression model.

**Results:**

227 subjects (110 non-DR and 117 DR) were selected in the analyses by using undersampling method to balance the sample size. Compared with non-DR group, DR group had higher DII values (1.14 ± 0.29 vs. 1.49 ± 0.21, *p* = 0.32), higher levels of HbA1c (6.8 ± 1.1% vs. 7.7 ± 2.6%, *p* < 0.001), longer duration of diabetes (6.52 ± 12 years vs. 14 ± 11 years, *p* < 0.001). The odds rate (OR) of DII for DR from the logistic regression was 1.38 (95%CI 1.06–1.81, *p* < 0.001). HbA1c, diabetes duration and obesity were important influencing factors, and their ORs were 1.81 (95% CI:1.31–2.50), 1.12 (95%CI:1.04–1.20), 4.01 (95%CI:1.12–14.32), respectively. In addition, the most important dietary indices for DR were different across males and females.

**Conclusions:**

The current study demonstrates that a higher DII is associated with an increased risk of DR in US adults. Considering diet as a modifiable factor, limiting pro-inflammatory diets or encouraging an anti-inflammatory diet may be a promising and cost-effective method in the management of DR.

## Introduction

Diabetic retinopathy (DR) is a major complication of diabetes mellitus (DM), and its overall prevalence accounts for 35% in diabetes patients worldwide [1, 2]. With the rising incidence of diabetes, DR continues to be a leading cause of vision loss in many developed countries [3]. The causal risk factors of DR include hyperglycaemia, hypertension, diabetes duration [2, 4]. The excessive generation of reactive oxygen species (ROS) due to hyperglycemia triggers localized inflammation, disrupts mitochondrial function, impairs microvascular integrity, and leads to cellular apoptosis. The accumulation of ROS, the onset of local inflammation, and cellular demise are intricately interconnected and significantly impact every stage of the pathogenesis of DR [5, 6]. Moreover, microvascular impairment gives rise to ischemia and localized inflammation, culminating in the development of neovascularization, macular edema, and neurodysfunction, ultimately resulting in irreversible long-term blindness. Diet can effectively influence inflammation, and an unhealthy diet is associated with the pathophysiology of diabetes [7, 8]. In diabetes, overexpression of pro-inflammatory proteins including C-reactive protein (CRP) and cytokines (IL-1β, IL-6, and TNF-α) contributes to chronic inflammation. Anti-inflammatory diets, such as a high fiber, fruit, and vegetable and low-fat intake, can reduce inflammatory markers and thus the risk of diabetes [9]. The Mediterranean diet (MedDiet) is renowned for its anti-inflammatory properties [10]. Research suggests that following a MedDiet can be beneficial for patients with diabetes. This diet promotes healthy blood sugar control by emphasising whole grains, fruits, and vegetables with a low glycemic index. These foods help prevent rapid spikes in blood sugar levels after meals. The dietary pattern beneficially modulates the gut microbiota and immune system [11]. In addition, dietary polyphenols found in the MedDiet have the potential to modulate the activity of nicotinamide adenine dinucleotide phosphate (NADPH) oxidase and mitigate oxidative stress and metabolic inflammation mediated by Nuclear Factor-kappa B (NF-κB) [12]. By contrast, pro-inflammatory diets, including a high consumption of red, processed meat, saturated or trans-fat and refined carbohydrates, typically represented by Western diets, are related to increased inflammatory markers. Western diets have been recognized as the major contributor to metabolic disturbances and the development of obesity-related diseases including type 2 diabetes, hypertension, and cardiovascular disease [9].

In 2014, Shivappa.et.al developed the dietary inflammatory index (DII) based on extensive literature about various dietary components and inflammatory biomarkers to provide a quantitative means for assessing the inflammatory potential of people’s diets. The DII score for each diet plan was calculated using the amounts of each of 45 dietary components that comprise the DII. Anti-inflammatory foods have a lower DII score while pro-inflammatory foods have a higher DII score [13]. Including a diet with a higher DII score, as indicated by a previous study, increased the odds of both diabetic kidney disease [14] and long-term all-cause and cardiovascular mortality [15]. Zhang et al. found that a higher DII score, corresponding to a more proinflammatory diet, was associated with a higher risk of gestational diabetes [8]. Incorporating a diet abundant in anti-inflammatory nutrients, such as one high in n-3 polyunsaturated fatty acids (PUFA) [16] and fiber [17], can significantly reduce the likelihood of developing DR in individuals with either type 1 or type 2 diabetes.

To the best of our knowledge, there were no studies exploring the relation between the DII and DR using National Health and Nutrition Examination Survey (NHANES) data. Therefore, this study aimed to explore the association between DII and DR in adults participating in NHANES 2005–2008.

## Methods

NHANES is an ongoing, nationally representative survey conducted in 2-year cycles, and its website provides the information about study design, interviews, demographics, dietary assessment, physical examination, and laboratory data in detail. The data were combined from NHANES 2005–2006 and 2007–2008, and 20, 497 individuals were included. This study samples were limited to participants aged ≥ 40 years who were eligible for fundus photography and had complete retinal imaging status [18]. Therefore, 14,922 participants were excluded and 5,575 were left.

**Fig. 1:**
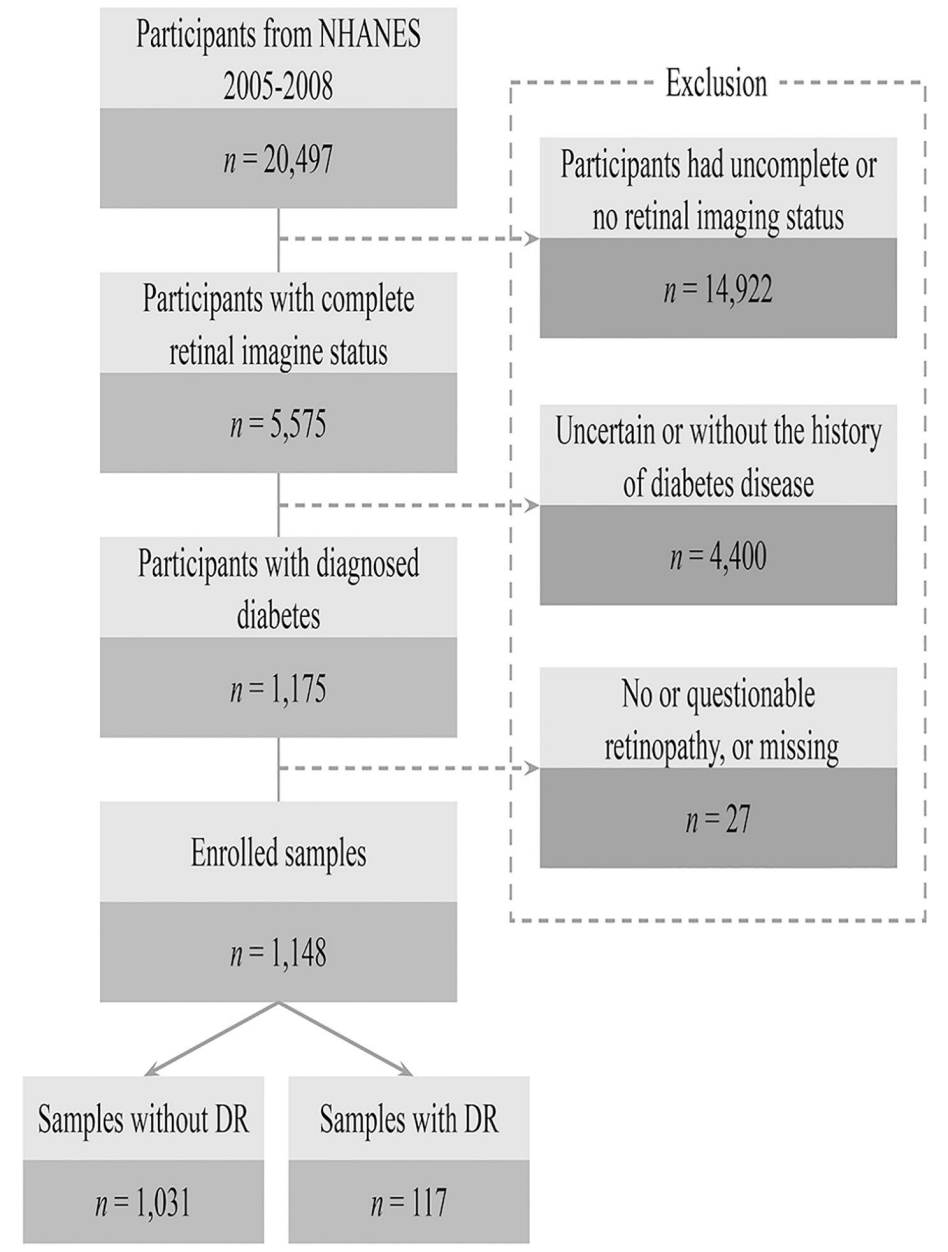
Flow chart of study participants. Sample selection and exclusion criteria for the comparison of DR and non-DR participants

Furthermore, individuals were classified as having diabetes if they (1) answered the self-reported diabetes status question, “Have you ever been told by a doctor or health professional that you have diabetes or sugar diabetes?”; (2) had a glycosylated hemoglobin A1c (HbA1c) value of at least 6.5%; (3) had a positive response to the question, “Are you now taking insulin?” or “Are you now taking diabetic pills to lower your blood sugar?” [17]. Among 5,575 individuals, there were 1,175 diabetes patients. Diagnosis of DR was based on the severity of DR of the worse eye. 27 individuals having (1) non-diabetic retinal disease specific retinopathy; (2) questionable retinopathy; (3) or missing data of retinopathy level were excluded from the analysis. Finally, 1,148 eligible individuals with 117 samples of DR and 1,031 samples of non-DR were selected for this analysis (see Fig. 1). Ethics approval was accepted by the institutional review board of the National Center for Health Statistics (NCHS) and study design was confirmed in accordance with the Helsinki Declaration. All participants provided informed consent before enrollment.

The present study calculated DII using the same method as Shivappa N et al. [13] which developed the method for calculating the DII based on dietary data. Dietary data of NHANES was collected by 24-hour dietary recall at Mobile Examination Centers (MEC), which was a private interview room which contained a standard set of measuring guides. These tools were used to help the respondent report the volume and dimensions of food items consumed. A second dietary recall was collected by telephone 3 to 10 days later to obtain a more complete picture of the usual dietary intake for all participants. In this study, out of 45 possible foods, 27 nutrients were used to calculate the DII score: carbohydrates; fat; protein; fiber; cholesterol; vitamins A, B1, B2, B6, B12, C, D, E; niacin; saturated, monounsaturated, and polyunsaturated fatty acids; omega3 and omega6 polyunsaturated fatty acids; iron; magnesium; zinc; selenium; folic acid; beta-carotene; alcohol; and caffeine. Previous studies indicated that 27 or 28 of nutrients applied for the calculation would not affect the DII predictive capacity [19, 20].

Selected covariates include age (years), sex (male/female), race (Mexican American/Non-Hispanic white/Non-Hispanic black/Other Hispanic/Other race/multi-racial), body mass index (BMI) (kg/m^2^), HbA1c (%), current smoking status (non-smoking/smoking), hypertension (yes/no), diabetes duration (years) and total energy intake 24-hour period prior to the interview (kcal) [17]. The BMI (kg/m^2^) levels were categorized as normal weight (less than 25), overweight (greater than or equal to 25 and less than 30) and obese (greater than or equal to 30). All the miss rates of covariates were lower than 45%.

Considering the complex survey design in NHANES, each sample was assigned a weight to measure the number of people in the population represented by that sample person. In the present study, according to the tutorials of NHANES, half of MEC exam weight determined the final weight since MEC examined subjects were a subset of those interviewed in the survey and two survey cycles were combined. All the statistical analyses of the present study were under weighted case. The two-tailed significance level 0.05 was considered in all analyses. The missing values were imputed by k-Nearest Neighbor (kNN) method in the package “DMwR2”. Then the DII scores of participants were calculated via standard approach. The resulting population was unbalanced, with 1031 non-DR individuals and 117 DR individuals. We adopted “ROSE” package for undersampling to balance the sample size of individuals in case group and control group [14]. After undersampling we obtained the final population of 227 individuals with 110 non-DR individuals and 117 DR ones.

All the descriptive statistics of continuous variables and categorical variables were calculated under weighted case. Mean and standard deviation were used to describe central tendency and dispersion if the continuous variable was normally distributed, otherwise median and interquartile range (IQR) were used. The differences of continuous variables between case and control group were performed by weighted two sample t-test since heteroscedasticity exists. The counterpart of categorical variables was conducted by weighted Chi-square test or Fisher’s test. Multivariate analysis was implemented by weighted Logistic regression with quasibinomial family in ‘survey’ package. The discretization of the DII score was executed by k-means clustering method.

## Results

After undersampling, there were 227 subjects (110 non-DR and 117 DR individuals) enrolled in our analysis. Account for the sample weight in NHANES, these 227 subjects actually represented 2,720,185 US adults with summing their weights. Their characteristics (all, non-DR and DR) are displayed in Table 1. The mean age of DR patients was 60.42 years. Compared with non-DR individuals, those with DR had higher DII scores (1.14 ± 0.29 vs. 1.49 ± 0.21, *P* = 0.32), higher level of HbA1c (6.80 ± 1.10% vs. 7.70 ± 2.60%, *P* < 0.001) and longer duration of diabetes (6.52 ± 12.00 years vs. 14.00 ± 11.00 years, *P* < 0.001).

**Table 1:** Demographics and characteristics of participants, NHANES 2005–2008

	Total	Non-DR	DR	P
Number of subjects, N (%)	227 2,720,185	110 (48.46) 1,371,497(50.42)	117 (51.54) 1,348,688(49.58)	
Age in years, mean (SD)	60.42 (0.93)	60.81 (1.35)	60.03 (1.29)	0.68
Sex, N (%)				0.94
Female	1,324,619(48.70)	663,065 (48.35)	661,554 (49.05)	
Male	1,395,566(51.30)	708,431 (51.65)	687,135 (50.95)	
Race, N (%)				0.10
Mexican American	312,414 (11.49)	134,081 (9.78)	178,333 (13.22)	
Non-Hispanic white	1,602,475(58.91)	914,609(66.69)	687,866 (51.00)	
Non-Hispanic black	675,679 (24.84)	254,956 (18.59)	420,723 (31.20)	
Other Hispanic	76,262 (2.80)	49,025(3.57)	27,237 (2.02)	
Other race or multi-racial	53,354 (1.96)	18,825 (1.37)	34,528 (2.56)	
BMI, kg/m^2^, mean (SD)	32.63 (0.69)	31.78 (0.90)	33.48 (1.02)	0.21
BMI, kg/m^2^, N (%)				0.43
<25	406,313 (14.94)	257,522 (18.78)	148,791 (11.03)	
25-<30	706,528 (25.97)	341,290 (24.88)	365,238 (27.08)	
>=30	1,607,344(59.09)	772,685 (56.34)	834,659 (61.89)	
HbA1c, median (IQR)	7.20 (1.90)	6.80 (1.10)	7.70 (2.60)	< 0.01
Current smoking status, N (%)				0.26
Non-smoking	2,317,250(85.19)	1,126,526(82.14)	1,190,725(88.29)	
Smoking	402,935 (14.81)	244,971 (17.86)	157,964 (11.71)	
Hypertension, N (%)				0.75
No	787,453 (28.95)	379,018 (27.64)	408,435 (30.28)	
Yes	1,932,731(71.05)	992,478 (72.36)	940,253 (69.72)	
Diabetes duration, years, median (IQR)	11.87(12.30)	6.52 (12.00)	14.00 (11.00)	< 0.01
DII, mean (SD)	1.31 (0.18)	1.14 (0.29)	1.49 (0.21)	0.32
Energy, median (IQR)	1734 (919)	1827 (768)	1665 (1229)	0.95

The 110 subjects of non-DR was randomly selected from 1031 subjects of non-DR.

a: The difference across the group was tested by weighted Welch two sample t-test since heteroscedasticity exists

Table 2 presents characteristics of 2,720,185 US adults in different quartiles of DII. The scores ranged from − 3.82 to -0.59, -0.39 to 1.45, 1.48 to 2.99, 3.09 to 4.73 in Q1, Q2, Q3 and Q4 groups, respectively. Except for Q2, the DII of the DR group is higher than that of the non-DR group in Q1, Q3 and Q4 groups but without significance. The difference of HbA1c levels were observed in Q1-Q4 group between non-DR and DR individuals, and higher in DR groups (*P*_Q1, Q2, Q3, Q4_<0.05). Diabetes duration was also significantly different between non-DR and DR individuals in Q2-Q4 group, and longer in DR groups (*P*_Q2, Q3, Q4_<0.05). Smoking was more prevalent in DR patients in the Q3(11.60%)-Q4 (27.25%) than Q1 (2.13%) -Q2 (5.57%), although this difference is not statistically significant (*P* > 0.05).

**Table 2:** Characteristics of participants in different quartiles of DII score, NHANES 2005–2008

	Q1 (-3.82, -0.59)				Q2 (-0.39, 1.45)				Q3 (1.48, 2.99)				Q4 (3.09, 4.73)			
	Total	Non-DR	DR	P	Total	Non-DR	DR	P	Total	Non-DR	DR	P	Total	Non-DR	DR	P
Number of subjects, N (%)	38 (100) 594,544 (100)	21 (55.26) 344,164 (57.89)	17 (44.74) 250,381 (42.11)	-	56 (100.00) 732,815 (100.00)	29 (51.79) 387,939 (52.94)	27 (48.21) 344,875 (47.06)		63 (100.00) 774,824 (100.0)	24 (38.10) 315,216 (40.68)	39 (61.90) 459,608 (59.32)	-	70 (100.00) 618,002 (100.00)	36 (51.43) 324,177 (52.46)	34 (48.57) 293,825 (47.54)	-
Age in years, mean (SD)	56.60 (1.87)	57.86 (2.92)	54.87 (1.63)	0.38	61.82 (1.71)	61.26 (2.28)	62.02 (2.53)	0.82	60.39 (1.89)	59.41 (2.93)	61.06 (2.45)	0.67	62.73 (1.78)	64.77 (2.40)	60.48 (2.56)	0.23
Sex, N (%)				0.32				0.60				0.19				0.76
Female	216,914 (36.48)	94,743 (27.53)	122,172 (48.79)		277,236 (37.83)	132,387 (34.13)	144,849 (42)		382,064 (49.31)	195,352 (61.97)	186,712 (40.62)		448,404 (72.56)	240,583 (74.21)	207,821 (70.73)	
Male	377,630 (63.52)	249,421 (72.47)	128,209 (51.21)		455,579 (62.17)	255,553 (65.87)	200,026 (58)		392,760 (50.69)	119,864 (38.03)	272,896 (59.38)		169,598 (27.44)	83,594 (25.79)	86,004 (29.27)	
Race, N (%)				0.72				0.16				0.55				0.24
Mexican American	89,262 (15.01)	44,949 (13.06)	44,314 (17.7)		56,386 (7.69)	34,683 (8.94)	21,704 (6.29)		84,363 (10.89)	22,389 (7.10)	61,974 (13.48)		82,403 (13.33)	32,061 (9.89)	50,342 (17.13)	
Non-Hispanic white	413,551 (69.56)	249,365 (72.46)	164,187 (65.57)		438,345 (59.82)	283,335 (73.04)	155,010 (44.95)		484,883 (62.58)	217,468 (68.99)	267,415 (58.18)		265,696 (42.99)	164,441 (50.73)	101,254 (34.46)	
Non-Hispanic black	59,621 (10.03)	23,434 (6.81)	36,187 (14.45)		193,121 (26.35)	64,341 (16.59)	128,780 (37.34)		186,167 (24.03)	62,183 (19.73)	123,984 (26.98)		236,770 (38.31)	104,998 (32.39)	131,772 (44.85)	
Other Hispanic	13,284 (2.23)	7591 (2.21)	5693 (2.27)		18,534 (2.53)	5581 (1.44)	12,953 (3.76)		19,410 (2.51)	13,175 (4.18)	6235 (1.36)		25,034 (4.05)	22,678 (7.00)	2357 (0.80)	
Other race or multi-racial	18,825 (3.17)	18,825 (5.47)	0 (0.00)		26,428 (3.61)	0 (0.00)	26,428 (7.66)		0 (0.00)	0 (0.00)	0 (0.00)		8100 (1.31)	0 (0.00)	8100 (2.76)	
BMI, kg/m^2^, median (IQR)	33.60 (10.68)	28.94 (8.49)	35.43 (9.38)	< 0.01	29.66 (11.08)	30.62 (12.28)	29.22 (5.65)	0.14	33.87 (11.49)	31.31 (13.6)	34.28 (11.49)	0.56	31.54 (9.11)	31.44 (9.21)	33.63 (9.70)	0.34
BMI, kg/m^2^, N (%)				0.07				0.18				0.70				0.86
<25	64,134 (10.79)	64,134 (18.63)	0 (0.00)		148,217 (20.23)	101,916 (26.27)	46,301 (13.43)		99,516 (12.84)	50,710 (16.09)	48,807 (10.62)		94,447 (15.28)	40,763 (12.57)	53,684 (18.27)	
25-<30	160,336 (26.97)	127,911 (37.17)	32,425 (12.95)		259,161 (35.37)	85,183 (21.96)	173,978 (50.45)		121,591 (15.69)	35,268 (11.19)	86,322 (18.78)		165,441 (26.77)	92,928 (28.67)	72,512 (24.68)	
>=30	370,075 (62.25)	152,119 (44.20)	217,955 (87.05)		325,437 (44.41)	200,841 (51.77)	124,597 (36.13)		553,717 (71.46)	229,238 (72.72)	324,479 (70.6)		358,115 (57.95)	190,486 (58.76)	167,629 (57.05)	
HbA1c, median (IQR)	7.20 (2.00)	7.00 (1.80)	9.50 (3.60)	< 0.05	7.30 (2.30)	6.80 (1.40)	8.20 (2.90)	< 0.05	6.80 (1.30)	6.70 (1.00)	7.36 (2.00)	< 0.01	7.10 (1.70)	6.60 (1.00)	8.10 (2.00)	< 0.01
Current smoking status, N (%)				0.23				0.08				0.72				0.84
Non-smoking	560,511 (94.28)	315,476 (91.66)	245,036 (97.87)		621,119 (84.76)	295,462 (76.16)	325,657 (94.43)		694,568 (89.64)	288,285 (91.46)	406,284 (88.4)		441,051 (71.37)	227,304 (70.12)	213,748 (72.75)	
Smoking	34,033 (5.72)	28,688 (8.34)	5345 (2.13)		111,696 (15.24)	92,478 (23.84)	19,218 (5.57)		80,255 (10.36)	26,931 (8.54)	53,324 (11.6)		176,951 (28.63)	96,874 (29.88)	80,077 (27.25)	
Hypertension, N (%)				0.73				0.72				0.09				0.73
No	263,707 (44.35)	164,541 (47.81)	99,166 (39.61)		191,772 (26.17)	91,842 (23.67)	99,930 (28.98)		175,694 (22.68)	33,972 (10.78)	141,722 (30.84)		156,281 (25.29)	88,664 (27.35)	67,617 (23.01)	
Yes	330,837 (55.65)	179,623 (52.19)	151,214 (60.39)		541,043 (73.83)	296,098 (76.33)	244,945 (71.02)		599,130 (77.32)	281,244 (89.22)	317,886 (69.16)		461,722 (74.71)	235,514 (72.65)	226,208 (76.99)	
Diabetes duration, years, median (IQR)	10.00 (13.00)	6.00 (14.18)	10.00 (6.00)	0.66	14.00 (13.00)	8.38 (10.00)	20.00 (20.00)	< 0.01	11.87 (14.50)	3.00 (8.11)	14.00 (11.00)	< 0.01	10.00 (13.00)	7.00 (14.00)	13.00 (7.00)	< 0.05
DII, mean (SD)	-1.50 (0.25)	-1.74 (0.37)	-1.18 (0.14)	0.16	0.64 (0.09)	0.69 (0.14)	0.59 (0.11)	0.57	2.13 (0.08)	2.11 (0.11)	2.12 (0.11)	0.86	3.78 (0.06)	3.74 (0.09)	3.82 (0.09)	0.50
Energy, median (IQR)	2374 (1449)	1933 (1528)	3065 (1310)	0.59	1853.54 (725)	1850 (555)	2106 (832)	0.39	1589 (760)	1979 (947)	1531 (535)	0.99	1125 (829)	1172 (828)	1011 (572)	0.36

Abbreviation: DR: Diabetic Retinopathy; DII: Dietary Inflammatory Index; HbA1c: Glycated Hemoglobin A1c; SD: Standard Deviation; IQR: Interquartile Range; BMI: Body Mass Index

The regression results are presented in the Table 3. Likelihood ratio test (LRT) among all models were all statistically significant, which means that Model 3 outperforms the other two models in fitting the dataset. Our result showed that the association between DII and the increase of DR was 1.38 (95% CI: 1.06–1.81). Furthermore, the results also showed that HbA1c, diabetes duration and obesity were important influencing factors, and their odds ratios (ORs) were 1.81 (95% CI: 1.31–2.50), 1.12 (95% CI: 1.04–1.20), 4.01 (95% CI: 1.12–14.32), respectively.

**Table 3:** Logistic regression analysis of DII for DR in participants, NHANES 2005–2008

	OR	95% CI	P-values
*Model 1*			
DII score	1.10	(0.92, 1.31)	0.30
*Model 2*			
DII score	1.12	(0.92, 1.35)	0.26
Age, y	0.99	(0.96, 1.02)	0.63
Sex			
Female	reference		
Male	1.10	(0.50, 2.41)	0.81
BMI, kg/m^2^			
<25	reference		
25-<30	1.88	(0.68, 5.24)	0.22
>=30	1.82	(0.68, 4.82)	0.23
*Model 3*			
DII score	1.38	(1.06, 1.81)	0.017
Age, y	1.00	(0.96, 1.04)	0.93
Sex			
Female	reference		
Male	0.95	(0.40, 2.22)	0.89
Race			
Mexican American	reference		
Non-Hispanic white	0.58	(0.18, 1.88)	0.36
Non-Hispanic black	0.81	(0.28, 2.35)	0.7
Other Hispanic	0.30	(0.05, 1.66)	0.17
Other race or multiracial	1.40	(0.16, 11.93)	0.76
BMI, kg/m^2^			
<25	reference		
25-<30	3.63	(0.88, 15.05)	0.07
>=30	4.01	(1.12, 14.32)	0.032
HbA1c, %	1.81	(1.31, 2.50)	< 0.001
Current smoking status			
Non-smoking	reference		
Smoking	0.63	(0.23, 1.71)	0.36
Hypertension			
No	reference		
Yes	0.86	(0.34, 2.21)	0.75
Diabetes duration, y	1.12	(1.04, 1.20)	0.002
Energy	1.00	(1.00, 1.00)	0.13

Model 1 did not adjust for any covariates; model 2 adjusted for potential confounding factors: age, sex, BMI; and model 3 further adjusted race, HbA1c, current smoking status, hypertension, diabetes duration and energy

Abbreviation: DR: Diabetic Retinopathy; DII: Dietary Inflammatory Index; HbA1c: Glycated Hemoglobin A1c; SD: Standard Deviation; IQR: Interquartile Range; BMI: Body Mass Index

**Fig. 2:**
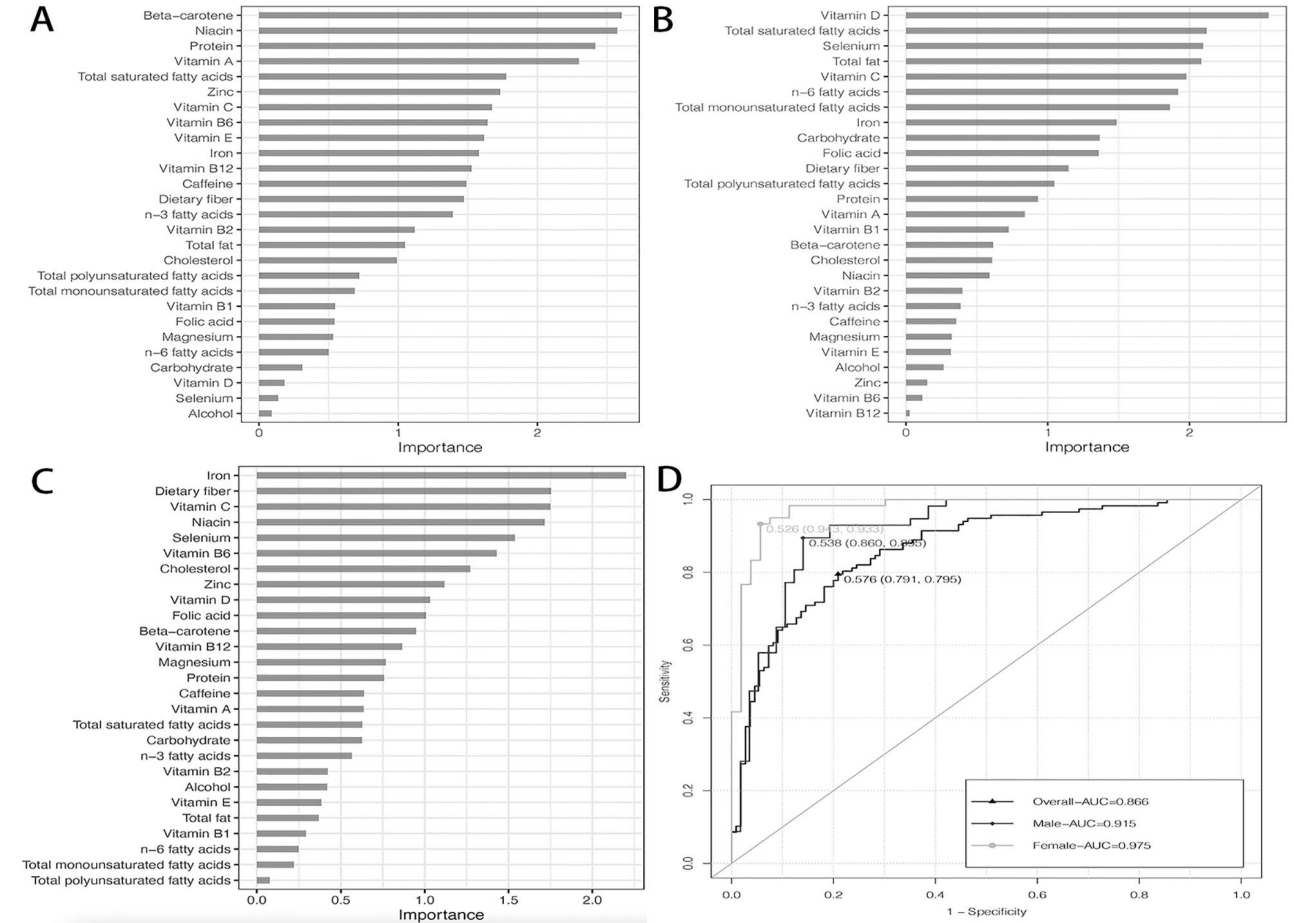
The most important indices and AUC curves in DR participants. (**A**) Beta-carotene, niacin, protein, total saturated fatty acids and vitamin A were important dietary indices for males with DR. (**B**) Vitamin D, total saturated fatty acids, selenium, total fat and vitamin C were important dietary indices for females with DR. (**C**) Iron, dietary fiber, vitamin C, niacin and selenium are the most important indices for total participants with DR. (**D**) AUC in male, female and total groups

We also distinguished important indices in DR subjects from non-DR subjects using logistic regression analysis with covariates. Beta-carotene, niacin, protein, total saturated fatty acids and vitamin A were important dietary indices for males (Fig. 2A). However, for females, they were vitamin D, total saturated fatty acids, selenium, total fat and vitamin C (Fig. 2B). Overall, the most important indices were iron, dietary fiber, vitamin C, niacin and selenium (Fig. 2 C). The areas under the curve (AUC) were 0.975, 0.915 and 0.866 for female, male and all groups, respectively (Fig. 2D).

## Discussion

The role of inflammation in the pathogenesis of DR is widely accepted [21–23]. However, there is currently a scarcity of research concerning the association between DII and DR. Notably, we showed that DII was positively associated with the increased risk of DR. HbA1c, diabetes duration and obesity were important influencing factors on multivariate analysis. Furthermore, dietary habits of males and females were also different.

Diet is one of the crucial factors which may cause systemic inflammation [24] and affect chronic low grade inflammatory status. For instance, cholesterol has shown a positive association with inflammation, whereas dietary fiber intake has been inversely associated [25]. The Mediterranean diet and the Western diet are renowned for their anti- and pro-inflammatory properties, as mentioned earlier [10]. Considering that DR is clearly connected with inflammation [26, 27], and DII serves as a tool to quantify the inflammatory potential of the diet, there is good reason to believe that DII is theoretically associated with DR. The present research is, as we know, the first thorough piece that investigated the connection between diet-related inflammation and DR in a cross-sectional study. Our results shows that a higher DII score may raise the risk of DR considerably. With 1 point increase in DII score, odds of having DR increased by 38%. It is inferred that a reasonable conclusion would be that an anti-inflammatory diet may play a positive role in preventing the occurrence of DR in DM patients.

Our research also demonstrated a strong connection between the development of DR and HbA1c level, the duration of DM and obesity. The higher mean HbA1c level, longer diabetes duration and obesity significantly elevate the risk of developing DR. In a study led by Harb W. and his team [28], it was discovered that among individuals diagnosed with type 2 DM, the duration of DM significantly varied across different categories of DR. Specifically, the average DM duration was notably longer in patients with mild non-proliferative DR (NPDR) (16.2 years), moderate NPDR (11.6 years), and proliferative DR (PDR) (13.2 years), in contrast to those without visible retinopathy (6.7 years) [28]. Coincidentally, patients with high HbA1c levels (OR:1.25; 95% CI, 1.18–1.32, per %), and those with a long duration of DM (OR: 1.10; 95% CI, 1.08–1.11, per year), are prone to DR in Singapore [29]. The aforementioned results are also supported by the findings of Almutairi et al. [30]. Obesity increases the susceptibility to type 2 diabetes, which is the predominant cause of DR [31]. The proportion of obese DM patients had a 4.01 times greater risk of DR compared to individuals with a normal BMI. These findings are consistent with previous research [32]. Furthermore, it’s noteworthy that the DII has been identified as being associated with all indicators of type 2 diabetes risk, including fasting glucose, insulin levels, and HbA1c, with obesity serving as a mediating factor [33].

Since diet has inflammatory potential, different dietary patterns may be associated with different chronic inflammatory diseases. For example, trans unsaturated fatty acid consumption raises the risk of cardiovascular disease and cardiovascular death [34]. Increased salt consumption may provoke water retention, thus leading to hypertension [35] On the contrary, dietary fiber is protective against colorectal cancer in patients in Asia [36]. The MedDiet may be a helpful technique for preventing coronary artery disease, heart failure outcomes, and metabolic problems [37]. Based on this, we believe that understanding the dietary structure of specific diseases is conducive to strengthening the cognition, management, and prevention of diseases. Therefore, our study distinguished important indices in DR subjects. Considering that there may be differences between male and female in life or diet patterns, work intensity, and social status, we conducted a sex subgroup analysis on dietary patterns. As we expected, there was a difference between the two groups. In addition to total saturated fatty acids, the male DR patients tended to prefer Beta-carotene, niacin, protein, and vitamin A diets, while the female DR patients tended to vitamin D, selenium, total fat, and vitamin C diets.

There were several strengths in our study. First, the k-means method was used to divide the data, which was more reasonable and effective for the discretization of continuous variables which were not suitable for clinical threshold and isometric grouping methods. Second, we also explored 27 components involved in the composition of DII to identify the dietary indices that played a major role in the occurrence of DR in different populations. This research also had certain limitations. First, since this was a cross sectional research, we were unable to account for the causal relationship between DII and DR. Second, the correlation between DII and the severity of DR was not discussed due to the sample size of patients in this study. Thirdly, it should be noted that due to sample size limitations, this article did not extensively delve into the role of sports activities. Additionally, the diagnosis of diabetes was based on self-reporting and did not include diabetes subtyping.

In conclusion, we demonstrated that the DII was strongly linked with DR in the US adult population. Higher DII scores may predict an increased occurrence of DR in multivariate analysis, but not in univariate analysis. We also discovered disparities in food patterns between males and females. Considering diet as a modifiable factor, limiting pro-inflammatory diets or encouraging anti-inflammatory diets might be a promising and cost-effective method in the management of DR.

## Data Availability

The datasets generated and analysed during the current study are available in online repositories. The names of the repository/repositories and accession number(s) can be found at: NHANES 2005–2006. https://wwwn.cdc.gov/nchs/nhanes/continuousnhanes/default.aspx?BeginYear=2005. NHANES 2007–2008. https://wwwn.cdc.gov/nchs/nhanes/continuousnhanes/default.aspx?BeginYear=2007.

## Abbreviations

NHANES: National Health and Nutrition Examination Survey
DII: Dietary Inflammatory Index
ROS: Reactive Oxygen Species
PUFA: Polyunsaturated Fatty Acids
MEC: Mobile Examination Centers
NCHS: National Center for Health Statistics
DR: Diabetic Retinopathy
CRP: C-reactive protein
BMI: Body Mass Index
OR: Odds Rate
VEGF: Vascular Endothelial Growth Factor
kNN: k-Nearest Neighbor
IQR: Interquartile Range
AUC: Areas Under the Curve
DM: Diabetes Mellitus
NPDR: Non-Proliferative Diabetic Retinopathy
PDR: Proliferative Diabetic Retinopathy
MedDiet: Mediterranean diet
HbA1c: Hemoglobin A1c
NF-κB: Nuclear Factor-kappa B
NADPH: Nicotinamide adenine dinucleotide phosphate
